# Improved *Plasmodium falciparum* dilution cloning through efficient quantification of parasite numbers and c-SNARF detection

**DOI:** 10.1186/s12936-021-03816-w

**Published:** 2021-06-23

**Authors:** Tatiane Macedo-Silva, Sanjay A. Desai, Gerhard Wunderlich

**Affiliations:** 1 Department of Parasitology, Institute for Biomedical Sciences, University of São Paulo, São Paulo, Brazil; 2 The Laboratory of Malaria and Vector Research, National Institute of Allergy and Infectious Diseases, National Institutes of Health, Rockville, MD 20852 USA

**Keywords:** Malaria, Plasmodium, Cloning, Limiting dilution

## Abstract

**Background:**

Molecular and genetic studies of blood-stage *Plasmodium falciparum* parasites require limiting dilution cloning and prolonged cultivation in microplates. The entire process is laborious and subject to errors due to inaccurate dilutions at the onset and failed detection of parasite growth in individual microplate wells.

**Methods:**

To precisely control the number of parasites dispensed into each microplate well, parasitaemia and total cell counts were determined by flow cytometry using parasite cultures stained with ethidium bromide or SYBR Green I. Microplates were seeded with 0.2 or 0.3 infected cells/well and cultivated with fresh erythrocytes. The c-SNARF fluorescent pH indicator was then used to reliably detect parasite growth.

**Results:**

Flow cytometry required less time than the traditional approach of estimating parasitaemia and cell numbers by microscopic examination. The resulting dilutions matched predictions from Poisson distribution calculations and yielded clonal lines. Addition of c-SNARF to media permitted rapid detection of parasite growth in microplate wells with high confidence.

**Conclusion:**

The combined use of flow cytometry for precise dilution and the c-SNARF method for detection of growth improves limiting dilution cloning of *P. falciparum*. This simple approach saves time, is scalable, and maximizes identification of desired parasite clones. It will facilitate DNA transfection studies and isolation of parasite clones from ex vivo blood samples.

## Background

Malaria remains a major health burden with about 405,000 deaths annually worldwide, with some 67% occurring in children under 5 years of age [1]. The most virulent human parasite, *Plasmodium falciparum*, accounts for nearly all these deaths. In vitro cultivation of *P. falciparum* has permitted increasingly sophisticated studies of parasite biology [2, 3]. During the last twenty years, many advances have been made using genetic manipulation of the parasite in the haploid blood stage phase [4]. Knockout, knockdown, and tagging of genes with degrons, ribozymes, mis-locators or posttranscriptional control of expression permitted functional testing of essential genes [5–8]. The introduction of CRISPR/Cas9 gene-editing approaches have accelerated genetic manipulation and enabled more rigorous studies of gene function [9].

Two persisting and major bottlenecks, however, are the low transfection efficiencies in *P. falciparum* and the subsequent effort and time-consuming process of isolating clonal transfectant parasites. For confident characterization of single genotypes, limiting dilution cloning and prolonged cultivation in microplates is required and is the generally accepted standard [10]. This method is also used for generating clones from ex vivo samples—e.g. clinical isolates or isolates from animals in genetic crosses.

Currently, parasite-infected cells are typically dispensed into 96-well microplates after dilution to less than 1 parasite/well and continuously cultivated with regular medium changes for 2–3 weeks. When the dilution is properly carried out, individual clones expand and are subsequently detected in a small number of microplate wells, yielding reliable clonal genotypic lines [10].

To ensure clonality and avoid seeding two parasites into any well, values of 0.2 to 0.3 infected cells/well are often selected. To obtain this low-titer dilution, workers must simultaneously estimate parasitaemia through microscopic examination of Giemsa-stained smears and the total number of red blood cells/unit volume using a hemocytometer cell counter [10, 11]. This method is laborious and subject to errors, which may be compounded through the need for two separate determinations. These errors are exacerbated when the parasitaemia is low, as is often the case in transfections where prompt dilution after outgrowth is needed to obtain slow-growing, desired integrands instead of parasites that carry only episomes. This concern also applies when slow-growing clones must be recovered from ex vivo isolates. A second problematic step in limiting dilution cloning is the identification of parasite growth in individual wells of the microplate. Most laboratories use microscopic examination of smears from each well of the microplate, an effort-intensive that cannot be scaled up to more than a few microplates. Thus, both inaccurate dilutions at the onset and failed detection of parasite growth in microplate wells can lead to failed recovery of an appropriate number of clones, leading to additional work and slowing scientific progress.

Here, an improved procedure for *P. falciparum* dilution cloning that combines precise flow cytometry-based parasite counts with facile detection of parasite outgrowth using the c-SNARF fluorescent pH indicator is presented [11].

## Methods

### Parasite culture and transfection

The *P. falciparum* NF54 and Dd2 laboratory lines were used in single homologous crossover recombination and CRISPR/Cas9 knock-in transfections. Blood-stage parasites were maintained in standard RPMI 1640-based media supplemented with 0.23% NaHCO_3_, 0.5% AlbuminNZ Microbiological BSA (MP Biomedicals, Irvine CA) and O^+^ human erythrocytes (UVA Blood Bank) under a defined gas atmosphere (90% N_2_, 5% O_2_ and 5% CO_2_) [2]. An identical, but HEPES-free culture medium was used with the c-SNARF pH indicator to facilitate detection of positive microplate wells. Parasite health was monitored by Giemsa-stained blood smears.

Schizont-stage NF54 cultures were used for single crossover recombination transfection using the protocol described by Hasenkamp and colleagues [12]. Beginning 2 days after transfection, 3 cycles of 2.5 nM WR99210 were applied for 15 days with intervening drug withdrawal for 15–20 days. The desired integration was then confirmed by PCR prior to initiating limiting dilution cloning. CRISPR/Cas9 knock-in transfection was performed using Dd2 parasites carrying a conditional Cas9-DDD cassette and the stabilizing agent trimethoprim (TMP). At day 2 after transfection, 2.5 µg/ml blasticidin S (Sigma) was added to select for a pL7 plasmid expressing sgRNA and a cassette for double homologous recombination knock-in. After regular media changes every 2–3 days, parasite outgrowth was detected by Giemsa smears 13–20 days after transfection. Limiting dilution cloning was then initiated.

### Flow cytometry

Flow cytometry was used to more precisely estimate parasitaemia and total cell count before limiting dilution cloning. In a typical experiment, 5 µl of packed cells from culture (with mostly trophozoites) was transferred to a sterile microfuge tube with 995 ul complete medium. After mixing, 500 µl of the suspension was transferred to a second tube.

To one microcentrifuge tube, either 0.5 µg/ml of ethidium bromide or SYBR Green I nucleic acid stain (2X dilution, Invitrogen) with 1xPBS was added to stain parasite DNA. After incubation for 20 min at 37 °C, these cells were washed twice with 500 µl 1xPBS before resuspension in 500 µl 1xPBS for flow cytometry using excitation and emission wavelengths of the laser lines of 488 nm for SYBR Green and 525 nm for ethidium bromide, reading the emitted fluorescence at 521 and 600 nm, respectively, with a 100,000 of event count. A matched sample of uninfected erythrocytes was used to identify the threshold for distinguishing infected cells from healthy ones. The flow cytometry readout was then used to estimate the number of cells/µl and percentage of infected cells. These values and the second microfuge of unstained cells from the original culture were then used to set up the limiting dilution.

### Parasite dilution and microplate culture

After determination of the number of parasites per µl by cytometry and a further dilution step in culture medium, a volume containing 20 parasites was transferred to a 15 mL Falcon tube; this volume ranged between 2 and 10 µl depending on the initial parasitaemia and haematocrit. Then, 9.5 ml complete medium and 0.5 ml fresh packed erythrocytes were added and mixed gently to establish a 5% final haematocrit. The solution was then dispensed in 100 µl aliquots in a 96-well plate using a multichannel pipet. Following this, the microplate was incubated for six days under the culture conditions described above. Medium changes occur on days 7, 11 and 14. From day 14 on, the plate was prepared for detection of positive wells by the method described by Lyko and colleagues [11]. Beginning on day 14, the medium was change to a HEPES-free medium containing 2 µM 5(and-6)- carboxy SNARF-1 (c-SNARF-1, Invitrogen, Carlsbad. CA. USA) [11].

### Detection of parasite growth in microplate wells with 5-(and-6)-carboxy SNARF-1

Microplates were typically incubated in HEPES-free medium containing c-SNARF-1 for 48 h to permit accumulation of metabolic acid in wells containing viable parasites. Microplates were removed from the incubator and allowed to equilibrate at room temperature for 15 min before measuring c-SNARF-1 fluorescence (excitation 488 nm, emission 590 and 645 nm; Synergy HT plate reader, BioTek, Winooski, VT, USA).

Wells with a significant increase in the 590/645 nm emission ratio were then expanded as clonal cultures and a maximum of 30 positive wells/plate was required to ensure clonality. Depending on the parasite growth rate, positive wells were detected as early as 16 days after initiating limiting dilution; slow-growing transfectants typically required longer. Calculation of the 590/645 nm fluorescence ratio and identification of positive wells were automated using scripts written in SigmaPlot 10.0 (Systat, San Jose, CA). These SigmaPlot scripts are available upon request.

## Results and discussion

### Parasite dilution after flow cytometry data shows an improved hit rate

**Fig. 1:**
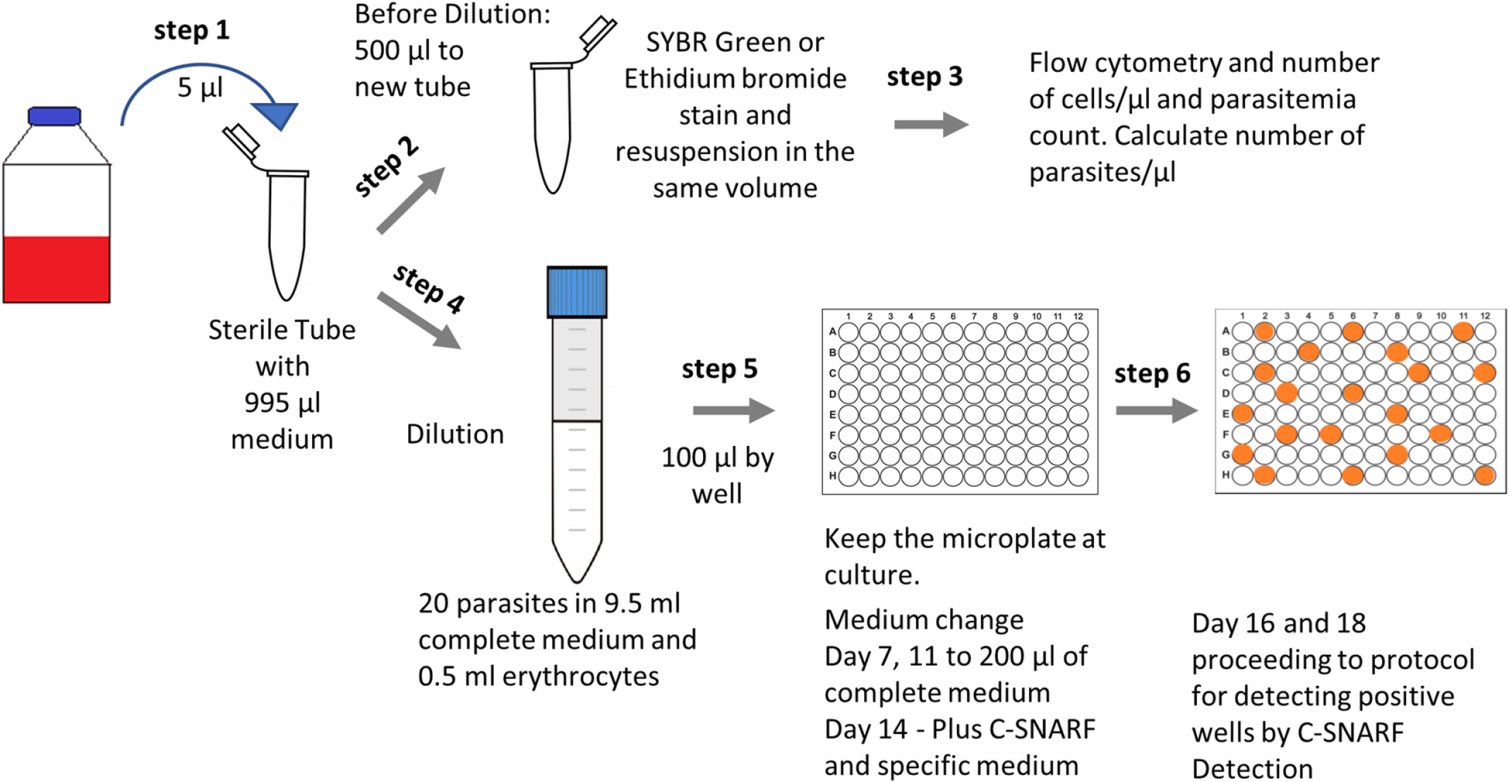
Schematic representation of steps using C-SNARF detection for *P. falciparum* cloning limiting dilution. In step 1, 5 µl of the medium-depleted cultured red blood cells are removed and diluted in 995 µl RPMI, and the parasitaemia is determined by flow cytometry using DNA stain EtBr or equivalent (step 2 and 3). After calculation of the total number of number of parasites per µl, a microliter volume corresponding to 20 parasites (dilution may be done stepwise) is mixed to result in 9.5 ml of a complete medium to which 0.5 ml of fresh blood is added (step 4). In sequence, 100 µl of this suspension is dispensed in 96 wells and the plate is then kept under the appropriate atmosphere and medium is changed on day 7, increased on day 11 to 200 µl and on day 14, the medium is changed to HEPES-free medium plus C-SNARF (step 5). At day 16 and 18 positive wells are detected by C-SNARF fluorescence (step 6)

To compare flow cytometry and different DNA staining methods in limiting dilution cloning experiments after transfection, the methods outlined in Fig. 1 were applied, establishing three experimental groups. In group A, parasitaemia and cell numbers per unit volume were determined using the traditional method with Giemsa-stained smears and a haemocytometer cell counter to obtain a desired parasite load of 0.3 parasites/well. Group B employed flow cytometry and ethidium bromide staining of parasite DNA to achieve an estimated 0.3 parasites/well. Group C also used flow cytometry, but detected parasites with SYBR Green I staining and dilution to 0.2 parasites/well in limiting dilution microplates (Table 1). Assuming random sampling with Poisson distribution, dilution to 0.2 and 0.3 parasites/well will yield growth of one or more parasites in 18% and 26% of wells, respectively. These values will lead to nonclonal lines that result from two or more parasites/well in 9.7% and 14.3% of positive wells, respectively. Both of these rates are acceptable [14].

**Table 1:** Detection of parasite containing wells per group transfected group

	Plate number	Positive wells	Detected modified/integrated loci
Group A	Plate 1	8	0
	Plate 2	42	Invalidated
	Plate 3	24	2
Group B	Plate 1	26	1
	Plate 2	32	2
	Plate 3	24	2
Group C	Plate 1	18	12
	Plate 2	16	Not tested
	Plate 3	21	Not tested

Group A represents three experiments that used manual determination of parasitaemia and total cell numbers with an estimated 0.3 parasites/well in limiting dilution microplates. Group B consists of three experiments using limiting dilution setup using flow cytometry with EtBr-DNA staining and 0.3 parasites/well. Group C consists of three trials of limiting dilution using flow cytometry, the SYBR Green I reagent, and 0.2 parasites/well. The column “Detected Modified/integrated Loci” shows the number of detected modified loci in each plate

**Fig. 2:**
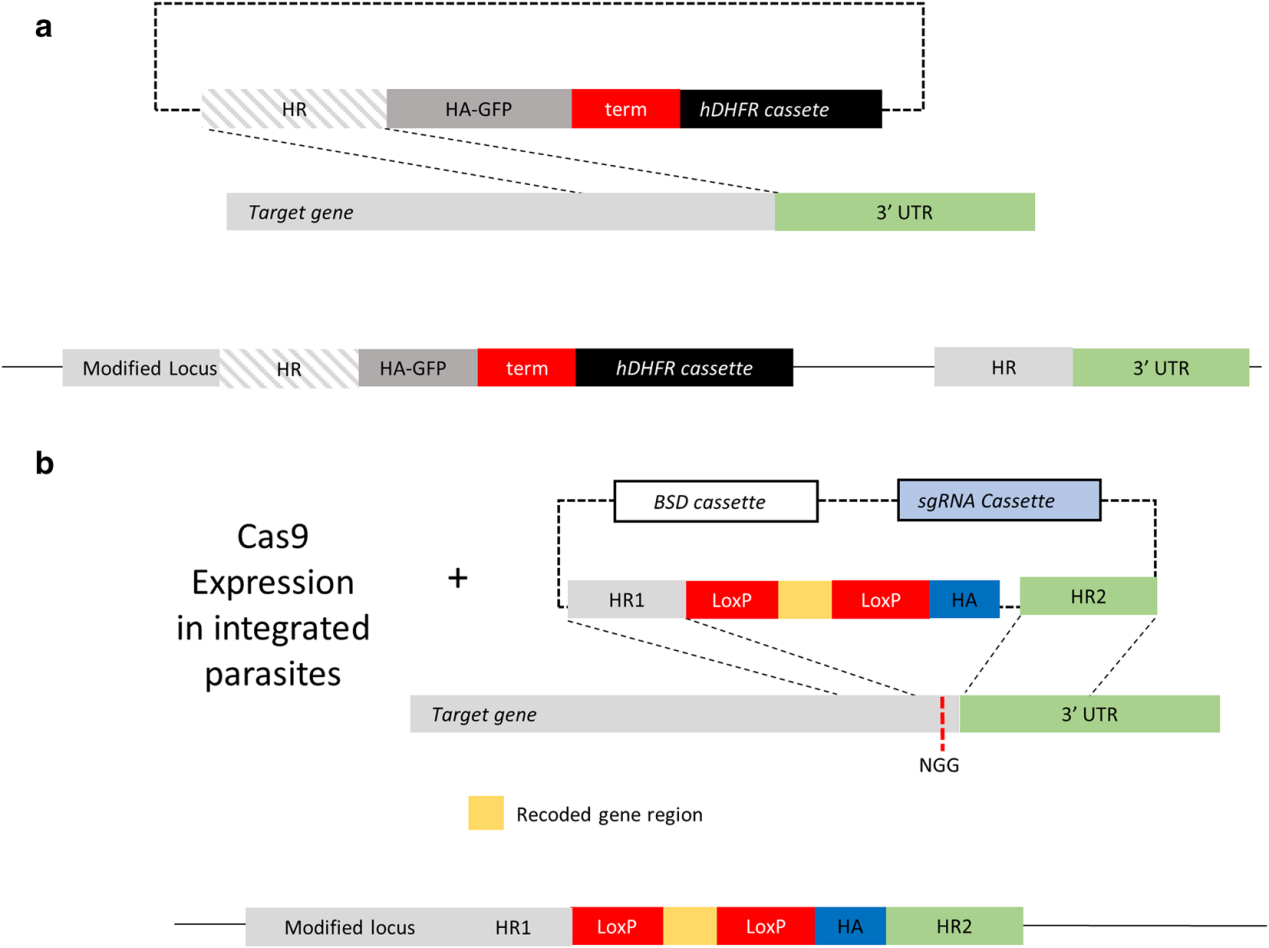
Plasmid constructs used in transfections. In A, the proposed model for single crossover recombination of the plasmid gene-GFP-HA is shown. The HR (homology region) consists of a sequence of 1013 bp, 76% A/T and the transfectant was selected using an hDHFR cassette. In B, the proposed model for a CRISPR/Cas9 transfection for insertion of a LoxP and a HA tag sequence into the target open reading frame (ORF). The transfection was performed into a parasite line already expressing Cas9 and the selection was done using blasticidin (2.5 µg/ml). HR1 (homolog region 1) consists of a target region in the coding region of the target gene (about 270 bp with 72% A/T) and HR2 target localizes in the 3’ untranslated region (3′ UTR); the size of HR2 was about 280 bp with 87% A/T. The red dashed line represents the NGG PAM site where Cas9 cleaves, mediated by the specific sgRNA. The yellow box represents the recodonized sequence which substitutes the original sgRNA target sequence to prevent continued cleavage of Cas9

The results for Group A, which relied on manual counting before each experiment, were highly variable, with 8, 42, and 24 positive wells from three is substantial higher compared with the other groups (Table 1). Notably, PCR checks of the 8 positive wells did not yield clones with the desired integration-positive genotype in this single crossover recombination experiment (Fig. 2A). The second plate, with 42 positive wells, was considered invalid due to a high probability of seeding two or more parasites per well and, therefore, mixed, non-clonal parasites. Of the 24 positive wells recovered in plate 3, only two were integration-positive. Thus, three plates were required to obtain the desired transfectant with this approach.

Group B yielded more reproducible results, with 26, 32, and 24 positive wells from the three trials (Table 1). PCR checks revealed 5 integration-positive clones from these plates, which also used single crossover recombination (Fig. 2A).

Group C, using CRISPR/Cas9 transfection (Fig. 2B) with flow cytometry and SYBR green staining to initiate dilution cloning with 0.2 parasites/well, yielded 18, 16, and 21 positive wells from three trials for a low standard deviation. PCR revealed that 12 of 18 tested positive wells yielded the desired integrant, consistent with efficient CRISPR/Cas9-mediated gene editing [9].

Thus, when a single crossover recombination approach is preferred, careful dilution and setup of limiting dilution and facile genotype determination of positive wells is especially critical. Selection-linked integration may be considered for knock-in experiments when CRISPR/Cas9-based methods are not suitable [7].

Efficient detection of parasites with both ethidium bromide and SYBR Green I staining was obtained and other DNA-staining dyes, such as Hoechst and Syto16, may also work. Ethidium bromide is the most cost-effective, but some laboratories restrict its use on flow cytometers as it is reported to be more mutagenic than other DNA stains. SYBR Green I also permits quantification of different parasite stages (ring, trophozoite and schizonts) due to its linear correlation of fluorescent signal with DNA content [13, 14].

The shown flow cytometry method can be completed in a few minutes to improve limiting dilution experiments, which may fail due to either an excessively low number of positive wells that do not yield desired clones or an excessively high number of positives that cannot be reliably considered clonal.

### Facile detection of positive wells with the C-SNARF method

**Fig. 3:**
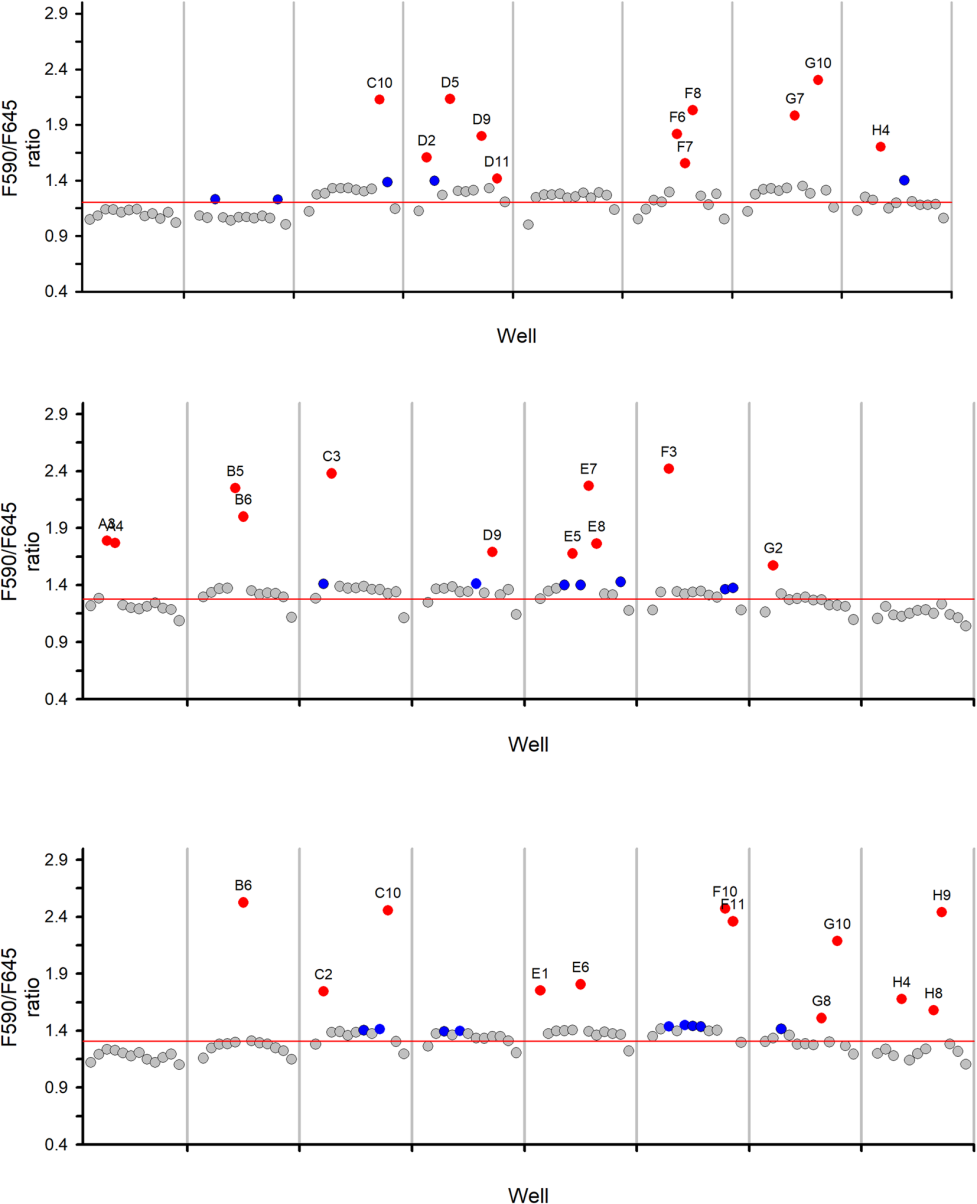
Parallel detection of positive wells with C-SNARF I and Giemsa-stained blood smears. The ratio for 590/645 nm C-SNARF fluorescence detected on 18 day from each of three limiting dilution microplates is shown for Group C. Red circles represent wells detected by C-SNARF on day 18; blue circles represent wells that were positive by blood smear only on day 18. Wells shown as grey circles were negative by both methods

Conventional detection of positive wells during limiting dilution cloning requires preparation and examination of 96 smears every 2 days to ensure that all clonal lines are identified and harvested. This process is both laborious and time-consuming. To evaluate whether positive wells can be more easily detected with the c-SNARF method [11], Group C transfections were subjected to detection with both microscopic examination and the pH-sensitive c-SNARF 1 dye. Figure 3 shows positive wells detected by both microscopic examination and an increased 590/645 nm fluorescence ratio on day 18 (red symbols). Some wells were identified as positives by microscopic examination, but did not exhibit an increased 590/645 nm fluorescence ratio on this day (blue symbols). Fluorescence measurements on these plates 2 days later revealed that these wells also became positive by the c-SNARF 1 method, consistent with a higher parasite load required for sufficient metabolic acidification to allow ratiometric detection. As these three plates yielded numbers of positive wells predicted from the initial setup at 0.2 parasites/well, leading to the conclusion that c-SNARF 1 is nontoxic to parasite cultures, as reported previously [11]. This avoids plate transfers as required for PCR and other methods, reducing cost and the risk of cross-contamination of wells. Although modestly longer cultivation of limiting dilution microplates is required for detection of positive wells, the c-SNARF method confidently identified all positive wells with dramatically reduced effort. In addition, it also permits scaling to larger numbers of microplates as positives may be identified from individual plates in ~ 2 min by most fluorescence plate readers.

## Conclusion

A streamlined approach for a precise setup of limiting dilution microplates for *P. falciparum* cloning based on flow cytometry and detection of positive wells with the c-SNARF method is reported. This approach is especially useful with low efficiency single crossover recombination transfections, where the fraction of desired integrant lines may be relatively low. It also saves time and reduces the risk of failed experiments with CRISPR/Cas9 transfections. When combined, flow cytometry-based setup and fluorescent detection of positive wells will enable rapid detection and propagation of clonal parasite lines.

## Data Availability

All data generated and analysed are described in this published article. The datasets used and analysed during the prototype study are available from the corresponding author on reasonable request. The SigmaPlot scripts used herein are available upon request (sdesai@niaid.nih.gov).

## References

[1] WHO (2019). World Malaria Report 2019.

[2] Trager W, Jensen JB (1976). Human malaria parasites in continuous culture. Science.

[3] Maier AG, Rug M (2013). In vitro culturing *Plasmodium falciparum* erythrocytic stages. Methods Mol Biol.

[4] Wu Y, Sifri CD, Lei H-H, Su X-Z, Wellems TE (1995). Transfection of *Plasmodium falciparum* within human red blood cells (malaria/chloramphenicol acetyltransferase/gene expression/codon usage). Proc Natl Acad Sci USA.

[5] Armstrong CM, Goldberg DE (2007). An FKBP destabilization domain modulates protein levels in *Plasmodium falciparum*. Nat Methods.

[6] Prommana P, Uthaipibull C, Wongsombat C, Kamchonwongpaisan S, Yuthavong Y, Knuepfer E (2013). Inducible knockdown of *Plasmodium* gene expression using the glmS ribozyme. PLoS One..

[7] Birnbaum J, Flemming S, Reichard N, Soares AB, Mesén-Ramírez P, Jonscher E (2017). A genetic system to study *Plasmodium falciparum* protein function. Nat Methods.

[8] Ganesan SM, Falla A, Goldfless SJ, Nasamu AS, Niles JC (2016). Synthetic RNA–protein modules integrated with native translation mechanisms to control gene expression in malaria parasites. Nat Commun.

[9] Ghorbal M, Gorman M, Macpherson CR, Martins RM, Scherf A, Lopez-Rubio J-J (2014). Genome editing in the human malaria parasite *Plasmodium falciparum* using the CRISPR-Cas9 system. Nat Biotechnol.

[10] Rosario V (1981). Cloning of naturally occurring mixed infections of malaria parasites. Science.

[11] Lyko B, Hammershaimb EA, Nguitragool W, Wellems TE, Desai SA (2012). A high-throughput method to detect *Plasmodium falciparum* clones in limiting dilution microplates. Malar J.

[12] Hasenkamp S, Russell KT, Horrocks P (2012). Comparison of the absolute and relative efficiencies of electroporation-based transfection protocols for *Plasmodium falciparum*. Malar J.

[13] Grimberg BT (2011). Methodology and application of flow cytometry for investigation of human malaria parasites. J Immunol Methods.

[14] Izumiyama S, Omura M, Takasaki T, Ohmae H, Asahi H (2009). *Plasmodium falciparum*: development and validation of a measure of intraerythrocytic growth using SYBR Green I in a flow cytometer. Exp Parasitol.

